# Pasos Hacia La Salud: a randomized controlled trial of an internet-delivered physical activity intervention for Latinas

**DOI:** 10.1186/s12966-016-0385-7

**Published:** 2016-05-28

**Authors:** Bess H. Marcus, Sheri J. Hartman, Britta A. Larsen, Dori Pekmezi, Shira I. Dunsiger, Sarah Linke, Becky Marquez, Kim M. Gans, Beth C. Bock, Andrea S. Mendoza-Vasconez, Madison L. Noble, Carlos Rojas

**Affiliations:** Department of Family Medicine and Public Health, University of California, San Diego, 9500 Gilman Drive, La Jolla, CA 92093-0628 USA; Department of Health Behavior, School of Public Health at University of Alabama at Birmingham, Birmingham, AL USA; Centers for Behavioral and Preventive Medicine, Department of Psychiatry and Human Behavior, Miriam Hospital, Providence, RI and Warren Alpert Medical School at Brown University, Providence, RI USA; Department of Behavioral and Social Sciences and the Institute for Community Health Promotion, School of Public Health, Brown University, Providence, RI USA

**Keywords:** Physical activity, Latinas, Internet, Technology, Behavioral intervention, Public health

## Abstract

**Background:**

Internet access has grown markedly in Latinos during the past decade. However, there have been no Internet-based physical activity interventions designed for Latinos, despite large disparities in lifestyle-related conditions, such as obesity and diabetes, particularly in Latina women. The current study tested the efficacy of a 6-month culturally adapted, individually tailored, Spanish-language Internet-based physical activity intervention.

**Methods:**

Inactive Latinas (*N* = 205) were randomly assigned to the Tailored Physical Activity Internet Intervention or the Wellness Contact Control Internet Group. Participants in both groups received emails on a tapered schedule over 6 months to alert them to new content on the website. The primary outcome was minutes/week of moderate to vigorous physical activity (MVPA) at 6 months as measured by the 7-Day Physical Activity Recall; activity was also measured by accelerometers. Data were collected between 2011 and 2014 and analyzed in 2015 at the University of California, San Diego.

**Results:**

Increases in minutes/week of MVPA were significantly greater in the Intervention Group compared to the Control Group (mean difference = 50.00, SE = 9.5, *p* < 0.01). Increases in objectively measured MVPA were also significantly larger in the Intervention Group (mean differences = 31.0, SE = 10.7, *p* < .01). The Intervention Group was also significantly more likely to meet national physical activity guidelines at 6 months (OR = 3.12, 95 % CI 1.46–6.66, *p* < .05).

**Conclusion:**

Findings from the current study suggest that this Internet-delivered individually tailored intervention successfully increased MVPA in Latinas compared to a Wellness Contact Control Internet Group.

**Trial registration:**

NCT01834287.

## Background

Ample evidence demonstrates the health benefits of physical activity (PA) and its role in the prevention of obesity, cardiovascular disease, diabetes, some cancers, and all-cause mortality [1]. However, PA levels for the large majority of Latinos are below U.S. national guidelines [2–8] and lower compared to non-Latino whites [5–8]. Both being Latino and being female are associated with not meeting PA recommendations [8, 9], and Latinas report the lowest levels of leisure PA of all major demographic groups. Concordantly, they also show marked disparities in obesity, diabetes, and other conditions related to inactivity [9, 10]. Therefore, effective interventions for Latinas, that have the potential for broad cost-effective dissemination, are needed.

Despite the great need to promote PA among Latinas, few interventions have targeted this specific population; a 2014 systematic review found 16 such interventions in a period of 30 years [11]. The majority of these utilized face-to-face delivery channels, such as site visits, church/community settings, or *promotora*-led walking groups. These approaches may be difficult for Latinas, who often cite limited transportation and childcare duties as key barriers to physical activity [11–13]. Such approaches may also be limited in their potential for widespread dissemination. Home-based PA interventions delivered through mediated channels have great potential for broader dissemination for the Latina population. Accordingly, a recent study showed that a PA intervention using printed, mail-delivered materials that were individually tailored based on theoretical constructs (Social Cognitive Theory and the Transtheoretical Model [14, 15]) was successful at increasing PA in inactive Latinas [16, 17].

While this study showed the intervention to be effective at increasing PA, the print-based, mail delivered format may not be the most efficient or cost-effective media channel for widespread dissemination. Recent data show that Internet access has grown markedly in Latinos during the past decade, with 83 % of Latinos reporting using the Internet regularly in 2014 (vs. 64 % in 2009) [18, 19]. Additionally, the largest gains were seen in foreign-born and Spanish-language dominant Latinos, who also tend to report the lowest rates of PA [9, 10, 20].

Given the potential of the web for broad dissemination, we recently adapted our effective Spanish language print-based PA intervention for the web. We conducted a series of focus groups with Latinas regarding their Internet use behaviors (i.e. why, when and how often they use the web, and the types of sites they visit), and used this information to build a web-based version of our intervention. We subsequently conducted a randomized controlled trial to determine the efficacy of the intervention relative to a Spanish-language Wellness Contact Control Internet Group. We hypothesized that Latinas randomized to the Tailored Physical Activity Internet Intervention Group would report significantly greater increases in minutes per week of moderate to vigorous physical activity (MVPA) from baseline to 6 months (post-treatment) than those in the Wellness Contact Control Internet Group. Findings from this study are described in this paper.

## Methods

### Design

The Pasos Hacia La Salud study (*N* = 205) was a randomized controlled trial of a 6-month Spanish-language, culturally and linguistically adapted, individually tailored, Internet-based Physical Activity Intervention, compared to a Spanish-language Wellness Contact Control Internet Group. The intervention was based on the Transtheoretical Model (TTM) and Social Cognitive Theory (SCT) [14, 15] and emphasized behavioral strategies for increasing activity levels, including goal-setting, self-monitoring, problem-solving barriers, increasing social support, and rewarding oneself for meeting physical activity goals. Data were collected between 2011 and 2014 and analyzed in 2015. The primary outcome was minutes per week of MVPA as measured by the 7-Day Physical Activity Recall (7-Day PAR). This measure was used in preliminary studies, and thus was used to determine statistical power for the current study. The level of power was set at 80 % a priori and was used to determine the number of participants needed given estimated effect sizes from our previous studies. Minutes of MVPA were also measured objectively using accelerometers, and this served as an additional primary outcome.

### Setting and sample

The study was conducted at the University of California, San Diego, and human subjects approval was obtained from the Institutional Review Board. Inclusion criteria included the following: self-identified as Hispanic or Latino (or of a group defined as Hispanic/Latino by the Census Bureau); self-reported insufficient physical activity (defined as participating in MVPA less than 60 minutes per week); 18–65 years of age; verified BMI <45 kg/m^2^; regular access to an Internet-connected computer through home, work, or their community (e.g., public library, community center, neighbor’s house); and willingness to be randomly assigned to either of the two study conditions.

Exclusion criteria included the following: unable to read or speak Spanish fluently; history of coronary heart disease (history of myocardial infarction or symptoms of angina), diabetes, stroke, orthopedic conditions which limit mobility, or any other serious medical condition that would make unsupervised physical activity unsafe (as determined by the Physical Activity Readiness Questionnaire); [21] current pregnancy or plan to conceive in the next year; planning to move from the area within the next year; hospitalization due to a psychiatric disorder in the past 3 years; taking medication that may impair physical activity tolerance or performance; and/or scoring less than 17 (i.e. inadequate functional health literacy) on the Short Test of Functional Health Literacy in Adults (STOFHLA) [22].

Sample size calculation was based on the assumption that we would have 80 % power for testing the null hypothesis that the intention to treat effect is zero, versus the two-sided alternative that the effect is different for those randomized to the Intervention Group versus those randomized to the Control Group. These estimates were based on the reported change in MVPA over 6 months amongst a subset of Latina participants who reported having Internet access in our previous individually-tailored, print-based study [16, 17].

### Protocol

A detailed description of study protocols can be found elsewhere [23]. Briefly, the primary modes of recruitment included paid ads on Craigslist.org, participant referrals, and advertising in local Spanish language newspapers, and at local churches, stores, and health-focused events. After potential participants were screened over the phone for eligibility, they attended an orientation session and completed the informed consent process. Participants returned for a second visit during which the following baseline measures were completed: height and weight, waist and hip circumference, blood pressure, and percent body fat. At this visit, participants also received an ActiGraph GT3X+ accelerometer, with instructions to wear the accelerometer during waking hours for 7 consecutive days. One week following the measurement visit, participants returned with the accelerometer and completed a 10-min treadmill walk intended as a demonstration of moderate physical activity. Additionally, they completed baseline self-report physical activity measures, including the 7 day physical activity recall interview, and were randomly assigned to one of two Spanish-language Internet-based conditions: Tailored Physical Activity Internet Intervention or Wellness Contact Control Internet Group. Group assignment was determined using a permuted block randomization procedure, with small random sized blocks. Randomization was stratified by TTM stage of change to ensure an equal distribution of treatment assigned across levels of motivational readiness for physical activity.

### Tailored physical activity internet intervention (Intervention Group)

Participants randomized to the Intervention Group received access to a study website including the following features: 1) self-monitoring of minutes of activity and steps; 2) goal setting with graphs to compare goals to recorded minutes; 3) message board to foster social support between participants; 4) “ask the expert” where participants could anonymously ask questions to a PhD level researcher; 5) online resources such as maps to create walking routes and free exercise videos. In addition, participants completed monthly questionnaires that generated automated tailored physical activity reports. These reports included information regarding: 1) current stage of motivational readiness for physical activity; 2) current self-efficacy; 3) cognitive and behavioral strategies associated with physical activity (processes of change); 4) how the participant compares to individuals who are physically active and meeting national guidelines of 150 min per week of MVPA [2] (normative feedback); 5) how the participant compares to her prior responses (progress feedback-provided after the first month); and 6) useful facts about physical activity, such as health benefits, stretching, and heart rate monitoring. The reports draw from a bank of more than 300 messages addressing different levels of these psychosocial and environmental factors affecting physical activity. In addition, they received an online manual that was matched to their motivational readiness for physical activity. The manual emphasized strategies for increasing PA, such as goal-setting, self-monitoring, problem-solving barriers, methods for increasing social support, and rewarding oneself for meeting physical activity goals.

Staff also reviewed physical activity informational pages on the website with the participant at baseline. This includes several ways to determine if they were exercising at moderate intensity: target heart rate; rating of perceived exertion; mile pace (15–20 min mile); and reference to the 10-min treadmill walk participants completed. Participants also received information on exercising safely and how to report an injury to the study. Lastly, the website provided links to several online and community resources.

The Intervention Group received email prompts to access the intervention website weekly during month 1, bi-weekly during months 2 and 3, and monthly during months 4–6, with new physical activity information tip sheets made available on this schedule. Participants received monetary incentives to complete the study requirements, including $10 each month for completing the online monthly questionnaires, $25 for attending the 6 and 12 month assessment visits, and a $50 bonus for attending both visits.

### Wellness contact control internet group (Control Group)

The Wellness Contact Control Internet Group received access to a Spanish language website with information on health topics other than physical activity. The web-based content focused on diet and other factors associated with cardiovascular disease risk and included information from a series on heart health developed for Latinos by the National Heart Lung and Blood Institute. Topics included: Cut Down On Salt and Sodium, Cut Down on Fat and Not on Taste, Learn Your Cholesterol Number, Stress Management, Kick the Smoking Habit, Protect Your Heart-Lower Your Cholesterol, and Prevent High Blood Pressure [24]. Participants in the Control Group received the same monetary incentives and the same number of email contacts on the same schedule as the Intervention Group. Control Group participants also logged into a website (separate from the intervention website) to complete monthly surveys on the previously described wellness topics.

### Measures

Demographics were assessed at baseline with a brief questionnaire assessing age, education, income, occupation, race, ethnicity, history of residence in the U.S., marital status, and acculturation [25]. The STOFHLA [22] was also administered at baseline to evaluate adult literacy in the health care setting.

The 7-Day Physical Activity Recall (7-Day PAR) was used to calculate the needed sample size for the study based on 80 % power using effect sizes from previous studies, and so served as the primary outcome measure [26, 27]. The 7-Day PAR is an interviewer-administered instrument that provides details about the types of activities engaged in and an estimate of weekly minutes of physical activity; it uses multiple strategies for increasing accuracy of recall, such as breaking down the week into daily segments (i.e., morning, afternoon, and evening) and asking about many types of activities, including time spent sleeping and engaging in moderate, hard, and very hard activity. All domains of activity are included, such as leisure, transportation, and occupational activity. To further enhance the accuracy of self-reporting, participants walked on a treadmill for 10 min at a moderate intensity pace (3–4 miles per hour) just prior to completing the 7-Day PAR at baseline and again at follow-up. The 7-Day PAR has been used across many studies on physical activity and has consistently demonstrated acceptable reliability, internal consistency, and concurrent validity with objective measures of activity [28–32]. Past research indicates that the 7-Day PAR is sensitive to changes in MVPA over time [29, 30] and has good test-retest reliability among Latino participants [33].

Accelerometer-measured physical activity (ActiGraph 3X+) served as an additional primary outcome measure. Accelerometers measure both movement and intensity of activity and have been validated with heart rate telemetry [34] and total energy expenditure [35]. Accelerometer data was processed using the ActiLife software, with a cut point of 1952 to establish the minimum threshold for moderate intensity activity [36]. Participants were asked to wear the accelerometer on their left hip for 7 days. Valid wear time was classified as 5 days of at least 600 min of wear time each day or at least 3000 min of wear time over 4 days. To be counted in the total minutes/week of activity, activity had to occur in ≥10-min bouts.

Psychosocial measures related to depression, social support, stress, and physical activity enjoyment and environment were also completed. The Center for Epidemiological Studies Depression Scale (CES-D) is a 10-question measure of depressive symptoms [37] that has been translated and validated across different ethnic groups, with internal consistencies of .87 and above in both English and Spanish [38, 39] and 0.83 in our sample, Social support for physical activity was examined in terms of support from friends and family members for physical activity. The 13-question measure has three sub-scales with acceptable internal consistencies (alphas range from 0.61 to 0.91, 0.87–0.88 in this sample) and good criterion validity [40]. The Perceived Stress Scale (PSS) [41, 42] examines the degree to which specific situations are deemed as stressful in the past week. The PSS is validated and has been used in many studies examining the association between stress and health and has an internal consistency of 0.86 in this sample [43]. The Physical Activity Enjoyment Scale (PACES) [44] assesses the level of personal satisfaction derived from physical activity participation. The measure has 18 items with high internal consistency (alpha = 0.96 and 0.94 in this sample) and criterion validity [44]. Neighborhood Environment Walkability Scale, Abbreviated (NEWSA) includes 54 items [45, 46] assessing various aspects of the built environment related to walking, neighborhood aesthetics, and traffic. Several studies have supported the test–retest reliability of the NEWS [47, 48] as well as its construct validity by reporting significant differences on some NEWS sub-scales between neighborhoods selected to differ on walkability [46, 47] and modest correlations between NEWS sub-scales and accelerometer and self-reported estimates of physical activity [49].

Three measures - stage of change, self-efficacy for physical activity, and the processes of change - were administered at baseline and on a monthly basis via the website, and used to help generate the tailored expert system feedback reports for the Intervention Group. The 4-item stage of change measure has demonstrated reliability (Kappa = 0.78; intra-class correlation *r* = 0.84) as well as shown acceptable concurrent validity with measures of self-efficacy and current activity levels [50]. The 40-item processes measure contains 10 sub-scales that address a variety of processes of activity behavior change. Internal consistency of the subscales ranged from .62 to .96 [51] in past studies, and .61–89 in the current study. Self-efficacy, or confidence in one’s ability to persist with exercising in various situations, such as when feeling fatigued or encountering inclement weather, was measured with a 5-item instrument [52] developed by Marcus and colleagues (alpha = .82 with alpha = 0.72 in this sample).

### Data analysis

Using a single linear mixed effects regression model, mean minutes/week of MVPA (as obtained from the 7-Day PAR and separately for accelerometers) at follow-up was regressed on time, treatment, and time x treatment, in order to assess between group differences in minutes/week of MVPA at 6 months (primary study outcome). As baseline characteristics were balanced between groups, no additional covariates were included in the model. The model specified included a random, subject-specific intercept, to account for repeated, correlated measures of the outcome within participant. Non-linear trends were assessed by including quadratic effects (for example) but ultimately not presented, as the linear model was superior in fit. All analyses were conducted on the intent to treat sample, with all randomized participants included in the analysis.

Since a likelihood-based approach was used for estimation of regression parameters, estimated effects made use of all available data without directly imputing missing outcomes. A similar modeling strategy was used for the second primary physical activity outcome, objectively measured MVPA obtained via accelerometer. Models of objectively measured MVPA were additionally adjusted for wear time (as a covariate) in this case. As a way of corroborating self-reported MVPA, spearman rank correlations were calculated at baseline and follow up (7-Day PAR vs. accelerometer).

We also assessed whether there were differences between groups in the percentage of participants meeting national guidelines for physical activity, defined as reporting at least 150 min/week of MVPA. Using a logistic regression model implemented with generalized estimating equations with robust standard errors, we assessed treatment effects on the odds of meeting guidelines at follow-up.

Using a similar modeling approach to that described for our primary outcome, we assessed effects of treatment on changes in psychosocial constructs over time, including all variables targeted by the intervention (self-efficacy, processes, social support, enjoyment). Unadjusted descriptives over time as well as adjusted mean changes from baseline (and standard errors) are presented.

All analyses were carried out in SAS 9.3 with significance level set a priori at α = 0.05.

## Results

**Fig. 1:**
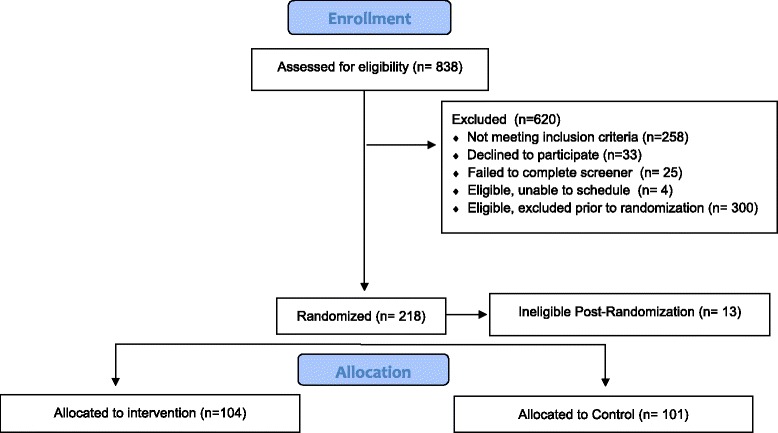
CONSORT flow diagram

The sample included 205 eligible women who were randomly assigned to the Intervention (*N* = 104) and Control (*N* = 101) groups, as 13 participants were deemed ineligible post-randomization. Reasons for not being randomized into one of the two conditions after signing a consent form included: medical condition that rendered the participant ineligible at time of assessment (e.g., high blood pressure), too much physical activity, inability to complete a treadmill demonstration, failure to attend scheduled visits, and no longer interested in the study. In addition, reasons for ineligibility post-randomization included unreliable computer access, moving away from San Diego, and medical issues that rendered participants ineligible (e.g., pregnancy, surgical procedures). Refer to Fig. 1. for the CONSORT diagram.

A comparison of between group differences in baseline demographics, activity level, psychosocial constructs and baseline measurements are presented in Tables 1 and 2.

**Table 1:** Demographic characteristics

Characteristics	Intervention (Mean and SD or %) (*N* = 104)	Control (Mean and SD or %) (*N* = 101)	Overall (M and SD or %) (*N* = 205)
Hispanic	100 %	100 %	100 %
Age	38.84 (10.61)	39.57 (10.36)	39.20 (10.47)
Generation Status in U.S. (% First) *N* = 204	86.5 %	77.0 %	81.9 %
BMI (kg/m2) *N* = 204	29.07 (5.82)	28.58 (4.50)	28.83 (5.20)
Race			
White	45.2 %	58.4 %	51.7 %
Mixed	17.3 %	14.9 %	16.1 %
Other	30.8 %	18.8 %	24.9 %
Ethnicity			
Mexican	82.7 %	86.1 %	84.4 %
Columbian	1.9 %	5.0 %	3.4 %
Guatemalan	1.9 %	0.0 %	1.0 %
Puerto Rican	1.0 %	1.0 %	1.0 %
Dominican Republic	1.0 %	0 %	0.5 %
Other	14.4 %	10.9 %	12.7 %
Yearly Household Income			
< $30,000	69.3 %	63.5 %	66.4 %
≥ $30,00 but < $50,000	17.3 %	24.7 %	21.0 %
≥ $50,000	9.6 %	6.9 %	8.3 %
Don’t Know	3.8 %	5.0 %	4.4 %
Employment Status			
Unemployed	49.0 %	41.0 %	45.1 %
Part Time	25.0 %	30.0 %	27.5 %
Full Time	25.0 %	29.0 %	27.0 %
Refused/Did Not Answer	1 %	0	0.5 %
Education Level (*N* = 204)			
< High School Graduate	14.6 %	13.9 %	14.2 %
High School Graduate	15.5 %	7.9 %	11.8 %
Vocational/Tech	14.6 %	11.9 %	13.2 %
≥ Some college	55.4 %	66.4 %	60.8 %
Language Spoken in the Home			
Only Spanish	40.4 %	34.7 %	37.6 %
More Spanish than English	30.8 %	32.7 %	31.7 %
Both Equally	15.4 %	23.8 %	19.5 %
More English than Spanish	11.5 %	5.0 %	8.3 %
Only English	1.9 %	4.0 %	2.9 %
Marital Status			
Married	50.0 %	57.4 %	53.7 %
Living with Partner	4.8 %	5.9 %	5.4 %
Separated	13.5 %	3.0 %	8.3 %
Divorced	10.6 %	16.8 %	13.7 %
Widowed	1.9 %	3.0 %	2.4 %
Never Married or Living with Partner	19.2 %	13.9 %	16.6 %
Health Literacy (scores of 23–26 “adequate”)	34.8 (2.7)	37.3 (22.8)	36.02 (16.13)

Data collected between 2011 and 2014 and analyzed in 2015 at University of California, San Diego

There were no between group differences, *p*’s > .05

**Table 2:** Baseline physical activity levels and related psychosocial variables (*N* = 205)

Variables	Intervention	Control	Overall
	(Mean and SD)	(Mean and SD)	(Mean and SD)
	(*N* = 104)	(*N* = 101)	
Self report MVPA (minutes/week, *N* = 205,)	8.01 (14.95)	10.44 (23.98)	9.20 (19.91)
Accelerometer measured MVPA in 10 min bouts (minutes/week, *N* = 200)	35.77 (69.65)	28.67 (48.22)	32.25 (59.96)
Self- Efficacy *N* = 200	2.27 (0.75)	2.40 (0.82)	2.34 (0.79)
Processes of Change, *N* = 205			
Cognitive Processes	2.42 (0.85)	2.49 (0.79)	2.45 (0.82)
Behavioral Processes	1.98 (0.64)	2.00 (0.58)	1.99 (0.61)
Social Support *N* = 202			
Friends Participation Score	15.17 (7.30)	14.67 (5.59)	14.93 (6.52)
Family Participation Score	17.59 (7.43)	17.96 (7.81)	17.77 (7.60)
Rewards and Punishments	3.50 (1.06)	3.36 (0.86)	3.43 (0.96)
Stage of Change, *N* = 205			
Precontemplation	6.7 %	5.0 %	5.9 %
Contemplation	74.0 %	76.2 %	75.1 %
Preparation	18.3 %	17.8 %	18.0 %
Action	1.0 %	1.0 %	1.0 %
Depression, *N* = 205	8.08 (5.65)	7.58 (5.55)	7.83 (5.59)
Enjoyment, *N* = 197	86.51 (21.68)	87.83 (18.75)	87.17 (20.22)
Stress, *N* = 201	22.97 (8.54)	22.18 (9.43)	22.58 (8.97)
Environment			
Residential Density, *N* = 205	250.14 (92.43)	228.95 (71.97)	239.70 (83.46)
Diversity, *N* = 130	2.87 (0.88)	2.91 (0.92)	2.89 (0.90)
Access, *N* = 204	3.34 (0.72)	3.27 (0.74)	3.31 (0.73)
Street Connectivity, *N* = 204	3.16 (0.70)	3.03 (0.80)	3.09 (0.75)
SWS, *N* = 204	2.86 (0.63)	2.98 (0.62)	2.91 (0.63)
Aesthetic, *N* = 205	2.74 (0.81)	2.74 (0.86)	2.74 (0.83)
Traffic, *N* = 205	2.29 (0.76)	2.17 (0.756)	2.23 (0.76)
Crime, *N* = 204	1.86 (0.83)	1.62 (0.77)	1.74 (0.81)

Data collected between 2011–2014 and analyzed in 2015 at University of California, San Diego

There were no between group differences, *p’s* > .05

Participants were 39.2 (10.5) years of age on average. The majority identified themselves as Mexican American (84.4 %), White (51.7 %) and first-generation in the U.S. (81.9 %). On average, participant BMI (28.8 +/− 5.2) was in the overweight range. Most participants had some college education (61 %) and had an annual household income lower than $30,000 (66.4 %). Participants reported low levels of physical activity at baseline, with mean self-reported min/week of MVPA of 9.2 (SD = 19.9) and objectively measured MVPA of 32.3 (SD = 60.0). There were no between-group differences in baseline characteristics, suggesting a successful randomization procedure.

The primary study outcome was self-reported MVPA at 6 month follow-up (adjusting for baseline values). On average, participants in the Intervention Group increased their min/week of MVPA from 8.0 (SD = 15.0), Median = 0 at baseline to 112.8 (SD = 97.1), Median = 100 min/week at 6 months compared to Control who reported 10.44(23.98) Median = 0 at baseline and 63.5 min/week (SD = 88.7), Median = 25 at follow-up. There was one statistical outlier at baseline (200 min/week of MVPA), which was removed from analysis. Adjusted model results showed a significant effect of Intervention vs. Control on min/week of MVPA, such that those randomized to the Intervention Group reported 50.0 more min/week of MVPA at 6 months compared to Control Group (adjusting for baseline), SE = 9.5, *p* < .01. See Table 3 for full regression model.

**Table 3:** Regression models corresponding to intervention effects on mean minutes/week of MVPA

	b	SE	*P*-value
Self-Reported MVPA			
Intercept	8.53	6.40	0.18
Intervention	−0.52	8.97	0.95
Time	54.54	9.00	<.001
Intervention*Time	50.26	12.85	<.001
Objectively Measured MVPA			
Intercept	28.58	6.80	<.001
Intervention	7.13	9.57	0.46
Time	15.49	9.19	0.09
Intervention*Time	23.90	13.04	0.05

Model run separately for two primary outcome variables. Effects reported here correspond to fixed effects from regression models and are considered unstandardized.

**Fig. 2:**
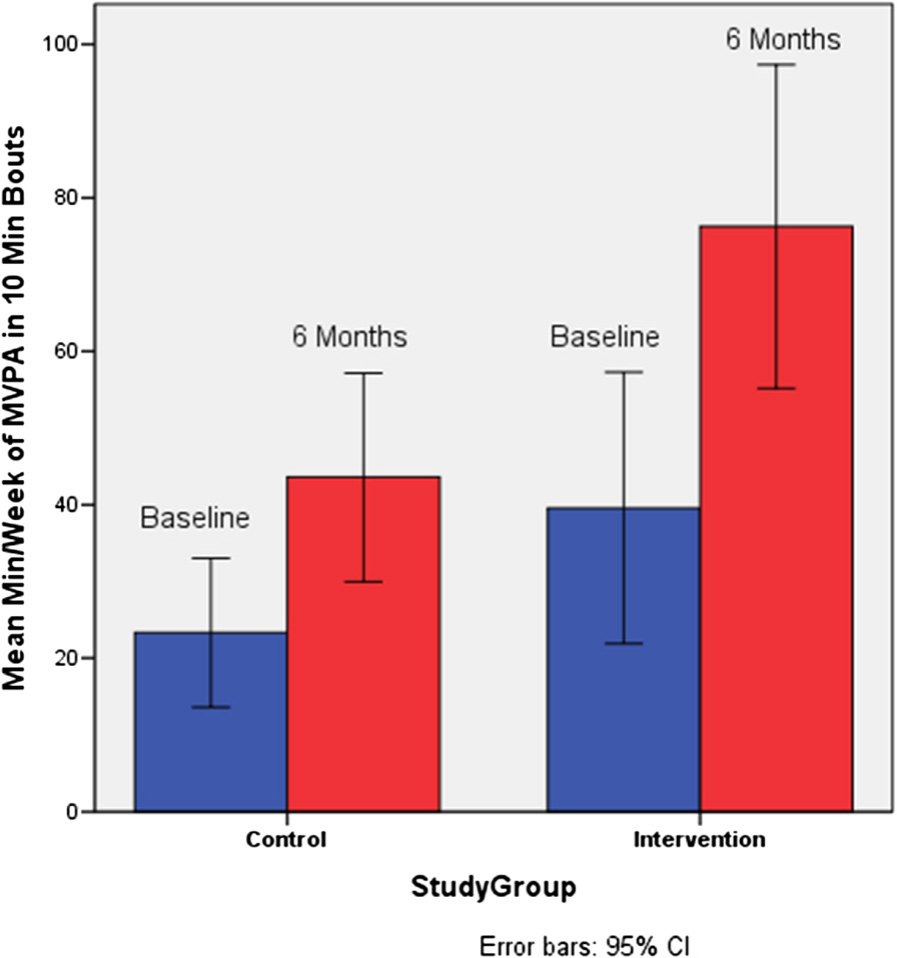
Unadjusted objectively measured MVPA over time by group

Unadjusted objectively measured MVPA over time is summarized in Fig. 2. Regression results indicate significant between-group differences in mean min/week of MVPA at 6 months, with significantly more minutes in the Intervention Group after controlling for baseline (mean differences = 31.0, SE = 10.7,*p* < .01). Results also show significant correlations between accelerometers and self-reported MVPA at baseline and 6 months (rho = 0.27, *p* < .01 at baseline and rho = 0.52, *p* < .01 at 6 months), Table 3.

Overall, 21.6 % of participants met national guidelines for PA at 6 months based on the 7-Day PAR: 31.4 % of the Intervention Group vs. 12.6 % of the Control Group. This corresponds to a statistically significant between group difference, OR = 3.12, 95 % CI: 1.46–6.66 (Table 4).

**Table 4:** Regression models corresponding to intervention effects on the probability of meeting ACSM criteria for Physical Activity

Reporting > =150 min/week of MVPA at 6 Months	b	SE	*P*-value
Intercept	−1.98	0.33	<.001
Intervention	1.14	0.38	0.003

Effects from regression models and are considered unstandardized

Unadjusted mean changes in targeted psychosocial constructs are presented separately by group in Table 5. Overall, adjusted results of changes from baseline to 6 months suggest Intervention participants showed greater increases in self-efficacy (.42 (1.03) vs. -0.13(0.92), *p* < .001), cognitive processes (0.64(0.95) vs. 0.05(0.79), *p* < .001), behavioral processes (0.92(1.00) vs 0.25(0.75), *p* < .001), and a trend for enjoyment (13.37(23.81) vs. 7.09(21.39),p = 0.08) from baseline to 6 months. There were no significant differences between Intervention and Control with respect to changes in social support (friends), social support (family), social support (rewards and punishments), perceived stress, or depression (*p’s* > .05).

**Table 5:** Unadjusted mean value of psychosocial constructs over time by group

	Intervention	Control
Self-Efficacy		
Baseline	2.27 (.75)	2.40 (.82)
**6 Months**	**2.71 (.96)**	**2.24 (.75)**
Behavioral Processes		
Baseline	1.97 (.64)	2.00 (.58)
**6 Months**	**2.86 (.90)**	**2.26 (.76)**
Cognitive Processes		
Baseline	2.42 (.85)	2.49 (.79)
**6 Months**	**3.00 (.88)**	**2.58 (.80)**
Social Support (Friends)		
Baseline	15.17 (7.30)	14.67 (5.59)
6 Months	17.05 (7.64)	15.72 (7.15)
Social Support (Family)		
Baseline	17.59 (7.43)	17.96 (7.81)
6 Months	21.46 (9.92)	19.78 (8.86)
Social Support (Rewards and Punishment)		
Baseline	3.50 (1.06)	3.36 (.86)
6 Months	3.67 (.99)	3.48 (1.17)
Enjoyment		
Baseline	86.51 (21.69)	87.83 (18.75)
6 Months	100.61 (19.45)	94.58 (21.79)
Depression		
Baseline	8.08 (5.65)	7.58 (5.55)
6 Months	10.88 (4.03)	10.98 (4.22)
Perceived Stress		
Baseline	22.97 (8.54)	22.18 (9.43)
6 Months	21.58 (8.57)	20.35 (9.48)

Mean (Standard Deviation). Bold data corresponds to significant between group differences in unadjusted means at given time (*p* < .05). Data collected between 2011–2014 and analyzed in 2015 at University of California, San Diego

## Discussion

Results from the current study support the efficacy of a Spanish-language individually-tailored Internet-based physical activity intervention for Latinas. The Intervention Group reported significantly greater increases in MVPA and several related psychosocial variables compared to the Control Group. A separate upcoming analysis of the maintenance effects is forthcoming. In the current study, the self-report physical activity data were validated with objective measures, which were significantly correlated with the 7-Day PAR and also showed a significant Intervention effect. The Control Group also reported increased physical activity at 6 months, which may have been due to repeat assessments of that variable. In addition, while the wellness materials focused on diet and other health behaviors aside from physical activity, it may have nonetheless inspired Control participants to engage in similar lifestyle changes. Social desirability response bias is another potential reason for increased MVPA in the Control Group.

These results are comparable to those found in a similar Internet-based physical activity study with mostly Non-Hispanic White participants, in which physical activity increased from a median of 0 min/week at baseline to 120 min/week at 6 months (vs. 0 median minutes/week at baseline to 100 min/week at 6 months in the current study) [52]. Also 44 % of the mostly Non-Hispanic White Intervention participants reported reaching the national physical activity guidelines (150 min/week) by 6 months, compared to 30.6 % in the current study with Latinas [53].

Findings from the current Internet-based study among Latinas were slightly more modest than those found in a similar study with mostly Non-Hispanic samples; however, increases in physical activity produced by the interactive web-based format used in the current study were greater than those found in a recent study in which similar content was provided to Latinas via mail-delivered self-help print materials [16, 17]. Specifically, self-reported physical activity increased from an average of 1.87 min/week (SD = 6.86) at baseline to 73.36 min/week (SD = 89.73) at 6 months among Intervention participants in the previous study, and only 11.36 % of the Intervention Group reported meeting national physical activity guidelines at 6 months in that study. Interestingly, while delivery channel seemed very important in the Latina samples, it was perhaps less critical to the mostly Non-Latino White participants, who reported similar physical activity levels at 6 months regardless of whether they received individually tailored interventions via Internet (median of 120 min/week) or print (112.5 min/week) [54]. Those findings were published in 2007, but more recent Health Information Trends Survey (HINTs) data also indicated that Latinos were more likely to use the Internet for help with diet, weight and physical activity than non-Latino whites [55]. Taken together, these findings suggest that the Internet is a particularly appealing delivery channel in this at-risk target population at this time.

Despite this, a 2013 Cochrane review reported a paucity of web-based physical activity intervention studies that include participants from varying socioeconomic or ethnic groups [56] and we were unable to locate such other studies in a recent literature review. Thus this likely constitutes the first application of interactive web-based technology for physical activity promotion among Latinas. Other strengths to the current study include the use of a randomized controlled trial research design, and balanced randomization across baseline characteristics.

As for limitations, this study was conducted with mostly healthy Mexican American women with some degree of health literacy and advanced education, and thus may not be generalizable to other Latina subgroups, Latino men, or other ethnic groups. Future studies should include formative research to determine how to modify the intervention for men and/or other ethnic groups. In addition, future could appeal to lower literate audiences by lowering the literacy level of the print portions of the website and/or changing some of the web content to video-based rather than print-based. Finally, while we included accelerometry as an additional primary outcome, the study was powered using a self-report measure.

Increases in MVPA in the current study could be seen as modest, with approximately one-third of the Intervention Group reaching the physical activity levels recommended for health benefits at 6 months. However, given the extremely low levels of MVPA at baseline, these gains in MVPA are encouraging, especially because getting completely inactive individuals to do some activity may be the most difficult and important change (compared to encouraging underactive individuals to meet guidelines). It may not be realistic to expect participants to go from sedentary to meeting guidelines within a 6-month period, thus future studies should focus on longer-term effects.

In addition, significant group differences were also found in the theoretic mediators directly targeted by the intervention, including greater self-efficacy and cognitive and behavioral processes of change among intervention participants compared to control participants. These psychosocial constructs have been shown to predict increases in PA in our prior studies with Latina women [16, 17]. To increase physical activity gains in future studies, we may need to influence relevant psychosocial variables such as social support and perceived stress that did not change in response to the current program. Social support in particular has been reported to be an important component in physical activity behavior change in past studies with Latinas [11, 57]. Further formative research could explore how websites can effectively improve social support for physical activity among Latinas and help more participants reach the national PA guidelines.

## Conclusions

Findings from the current study suggest that the Internet-delivered individually tailored intervention achieved even larger increases in physical activity than the print-based version used in our past studies with Latinas. This is an important finding given that the Latino population in the U.S. is rapidly growing and reports high rates of inactivity and related conditions (obesity, diabetes) [58]. To address these health disparities, this community needs appealing, effective physical activity interventions that can reach a large number of people in a cost-efficient manner. Unlike print-based interventions, web-based approaches can be offered to more people without substantially increasing the incremental cost of the intervention. Future researchers in this area are encouraged to focus on developing mobile friendly web sites as Latinas are frequently accessing the Internet via smartphones [20]. Other aspects of smart phone technology (applications, text messaging) have been used to promote health behavior change in other groups [59–62] and should also be explored in this at risk target population. Such features could help drive participants to the website and improve utilization, resulting in even greater behavior change.
